# Supplementary material for: Objective and Subjective Assessment of Music Perception and Musical Experiences in Young Cochlear Implant Users
Source: Audiol Res. 2024 Jan 18;14(1):86–95. doi: 10.3390/audiolres14010008 (PMC10801469; doi:10.3390/audiolres14010008)
Supplement: Supplementary file 1 [file audiolres-14-00008-s001.zip › audiolres-2756354-supplementary.pdf]

# SONGS FOR CHILDREN UNDER 10 YEARS

1. CUMPLEAÑOS FELIZ
2. CAMPANA SOBRE CAMPANA (VILLANCICO)
3. ESTRELLITA DONDE ESTÁS
4. SOL SOLECITO
5. EL PATIO DE MI CASA
6. BABY SHARK
7. SUÉLTALO (FROZEN 1)
8. EL COCHERITO LERÉ
9. CUCÚ CANTABA LA RANA
10. MUCHO MAS ALLÁ (FROZEN 2)
11. ESTABA EL SEÑOR DON GATO
12. UN ELEFANTE SE BALANCEABA
13. AL CORRO DE LA PATATA
14. CINCO LOBITOS
15. LOS PECES EN EL RÍO (VILLANCICO)

# SONS FOR ADOLESCENTS (11-16y)

(Name of the song and singer)

1. Canción de Bob esponja
2. De ellos aprendí (David Rees)
3. Chuchuwa (Cantajuegos)
4. Cumpleaños feliz (Parquís)
5. Teléfono (Aitana)
6. Sofía (Alvaro Soler)
7. Campana sobre campana (villancico)
8. Baby shark (Pinkfong)
9. Doraemon (Doraemon)
10. A Dios le pido (Juanes)
11. Madre tierra (Chayanne)
12. Suéltalo (Frozen1)
13. Resistiré (Duo Dinámico)
14. Si tú la quieres (Bisbal y Aitana)
15. Eso que tú me das (Jarabe de Palo)
16. Tusa (Niki Minaj)
17. Con altura (Rosalía)
18. Happier (Marshmello)
19. Hey Brother (Avicii)
20. Blinding lights (The weekend)
21. Play hard (David Guetta)
22. Stitches (Shawn Mendes)
23. Por primera vez (Camilo)
24. Danza kuduro (Don Omar y Lucenzo)
25. Waka waka (Shakira)
26. Roar (Katy Perry)
27. Hawaii (Maluma)
28. Tatoo (Rauw Alejandro)
29. One Kiss (Dua Lupa y Calvin Harris)
30. Súbeme la radio (Enrique Iglesias)

# Music questionnaire for pediatric population (MuQPP)

***NOMBRE*__________________________________________ *FECHA*________________**

## *INTERESES MUSICALES*

1 - ¿Te gusta escuchar música? NO☐ YA NO, ANTES☐ A VECES ☐ SÍ☐

2 - ¿Te gusta cantar? NO☐ YA NO, ANTES☐ A VECES ☐ SÍ☐

3 - ¿Tocas algún instrumento musical? NO☐ YA NO, ANTES☐ A VECES ☐ SÍ☐

4 - ¿Te gusta bailar? NO☐ YA NO, ANTES☐ A VECES ☐ SÍ☐

5 - ¿Te resulta fácil bailar al ritmo de la música? NO☐ YA NO, ANTES☐ A VECES ☐ SÍ☐

6 - ¿Te resulta fácil aprender una nueva melodía? NO☐ YA NO, ANTES☐ A VECES ☐ SÍ☐

7 - ¿Afecta la música a tu estado de ánimo? NO☐ YA NO, ANTES☐ A VECES ☐ SÍ☐

## *PERFIL MUSICAL*

8. ¿Cuántos días por semana escuchas música?

| 1 | 2 | 3 | 4 | 5 | 6 | 7 |
|---|---|---|---|---|---|---|
| ☐ | ☐ | ☐ | ☐ | ☐ | ☐ | ☐ |

9. ¿Con qué frecuencia <u>escuchas música</u> en las siguientes situaciones?

| | *Nunca* (1%) | *Raras veces* (12%) | *De vez en cuando (25%)* | *Generalmente* (50%) | *Frecuentemente* (75%) | *Casi* siempre (87%) | *Siempre* (99%) |
|---|---|---|---|---|---|---|---|
| 9.1.Cuando estoy sol@ en casa | 1☐ | 2☐ | 3☐ | 4☐ | 5☐ | 6☐ | 7☐ |
| 9.2.Cuando estoy acompañad@ en casa | 1☐ | 2☐ | 3☐ | 4☐ | 5☐ | 6☐ | 7☐ |
| 9.3.Cuando estoy con amig@s | 1☐ | 2☐ | 3☐ | 4☐ | 5☐ | 6☐ | 7☐ |
| 9.4.Cuando me voy a dormir | 1☐ | 2☐ | 3☐ | 4☐ | 5☐ | 6☐ | 7☐ |
| 9.5.Para relajarme | 1☐ | 2☐ | 3☐ | 4☐ | 5☐ | 6☐ | 7☐ |
| 9.6.Cuando hago deporte | 1☐ | 2☐ | 3☐ | 4☐ | 5☐ | 6☐ | 7☐ |
| 9.7.Mientras viajo (yendo al colegio…) | 1☐ | 2☐ | 3☐ | 4☐ | 5☐ | 6☐ | 7☐ |
| 9.8.Mientras estudio | 1☐ | 2☐ | 3☐ | 4☐ | 5☐ | 6☐ | 7☐ |

10. ¿Con qué frecuencia cantas?

| *Nunca* (1%) | *Raras veces* (12%) | *De vez en cuando* (25%) | *Generalmente* (50%) | *Frecuentemente* (75%) | *Casi todo* el tiempo (87%) | *Todo el tiempo* (99%) |
|---|---|---|---|---|---|---|
| 1☐ | 2☐ | 3☐ | 4☐ | 5☐ | 6☐ | 7☐ |

11. ¿Con qué frecuencia cantas en las siguientes situaciones?

| | *Nunca* (1%) | *Raras veces* (12%) | *De vez en cuando (25%)* | *Generalmente* (50%) | *Frecuentemente* (75%) | *Casi* siempre (87%) | *Siempre* (99%) |
|---|---|---|---|---|---|---|---|
| 11.1.Cuando estoy sol@ en casa | 1☐ | 2☐ | 3☐ | 4☐ | 5☐ | 6☐ | 7☐ |
| 11.2.Cuando estoy acompañad@ en casa | 1☐ | 2☐ | 3☐ | 4☐ | 5☐ | 6☐ | 7☐ |
| 11.3.Cuando estoy con amig@s | 1☐ | 2☐ | 3☐ | 4☐ | 5☐ | 6☐ | 7☐ |
| 11.4.Delante de otros | 1☐ | 2☐ | 3☐ | 4☐ | 5☐ | 6☐ | 7☐ |
| 11.5.Delante de una grabación | 1☐ | 2☐ | 3☐ | 4☐ | 5☐ | 6☐ | 7☐ |

## *PAPEL DE LA MÚSICA*

12. ¿Qué importancia tiene para ti la música en tu vida?

*Nada importante* *Muy importante*

1☐ 2☐ 3☐ 4☐ 5☐ 6☐ 7☐

## *ACTIVIDADES RELACIONADAS CON LA MÚSICA*

13. ¿Realizas alguna actividad musical (aparte de las clases de música en el colegio)? Por ejemplo: tocar la guitarra, el piano, teatro musical, tocar en un grupo musical…

SI ☐
NO ☐

Si has contestado que sí, ¿qué actividad realizas? ¿Y durante cuánto tiempo llevas haciéndola?

13.1. ☐ Grupo musical ___________años/meses

13.2. ☐ Coro musical ___________años/meses

13.3. ☐ Clases particulares de algún instrumento o de voz ___________años/meses

13.4. ☐ Tocar por diversión yo sol@ ___________años/meses

13.5. ☐ Tocar por diversión con amig@s ___________años/meses

13.6. ☐ Otra: ____________________________________________________________ ___________años/meses

- Si la actividad no la realizas actualmente, ¿la has realizado hace tiempo?

# Music questionnaire for pediatric population (MuQPP)

## *MUSICAL INTERESTS*

1 - Do you like listening to music? NO☐ NOT ANY MORE, BUT IT USED TO BE ☐ SOMETIMES ☐ YES☐

2 - Do you like singing? NO☐ NOT ANY MORE, BUT IT USED TO BE ☐ SOMETIMES ☐ YES☐

3 - Do you play a musical instrument? NO☐ NOT ANY MORE, BUT IT USED TO BE ☐ SOMETIMES ☐ YES☐

4 - Do you like dancing? NO☐ NOT ANY MORE, BUT IT USED TO BE ☐ SOMETIMES ☐ YES☐

5 - Do you find it easy to dance to music? NO☐ NOT ANY MORE, BUT IT USED TO BE ☐ SOMETIMES ☐ YES☐

6 - Do you find it easy to learn a new melody? NO☐ NOT ANY MORE, BUT IT USED TO BE ☐ SOMETIMES ☐ YES☐

7 - Does music affect your mood? NO☐ NOT ANY MORE, BUT IT USED TO BE ☐ SOMETIMES ☐ YES☐

## *MUSICAL PROFILE*

8. How many days a week do you listen to music?

| 1 | 2 | 3 | 4 | 5 | 6 | 7 |
|---|---|---|---|---|---|---|
| ☐ | ☐ | ☐ | ☐ | ☐ | ☐ | ☐ |

9. How often do you <u>listen to music</u> in the following situations?

| | *Never* (1%) | *Rarely* (12%) | *Occasionally* (25%) | *Mostly* (50%) | *Frequently* (75%) | *Almost always* (87%) | *Always* (99%) |
|---|---|---|---|---|---|---|---|
| 9.1. When I am alone at home | 1☐ | 2☐ | 3☐ | 4☐ | 5☐ | 6☐ | 7☐ |
| 9.2. When I am at home in company | 1☐ | 2☐ | 3☐ | 4☐ | 5☐ | 6☐ | 7☐ |
| 9.3. When I am with Friends | 1☐ | 2☐ | 3☐ | 4☐ | 5☐ | 6☐ | 7☐ |
| 9.4. When I go to sleep | 1☐ | 2☐ | 3☐ | 4☐ | 5☐ | 6☐ | 7☐ |
| 9.5. To relax | 1☐ | 2☐ | 3☐ | 4☐ | 5☐ | 6☐ | 7☐ |
| 9.6. When I do sports | 1☐ | 2☐ | 3☐ | 4☐ | 5☐ | 6☐ | 7☐ |
| 9.7. While travelling (going to school...) | 1☐ | 2☐ | 3☐ | 4☐ | 5☐ | 6☐ | 7☐ |
| 9.8. While studying | 1☐ | 2☐ | 3☐ | 4☐ | 5☐ | 6☐ | 7☐ |

10. How often do you sing?

| *Never* | *Rarely* | *Occasionally* | *Mostly* | *Frequently* | *Almost always* | *Always* |
|---|---|---|---|---|---|---|
| 1☐ | 2☐ | 3☐ | 4☐ | 5☐ | 6☐ | 7☐ |

11. How often do you <u>sing</u> in the following situations?

| | *Never (1%)* | *Rarely (12%)* | *Occasionally (25%)* | *Mostly* (50%) | *Frequently* (75%) | *Almost always* (87%) | *Always* (99%) |
|---|---|---|---|---|---|---|---|
| 11.1. When I am alone at home | 1☐ | 2☐ | 3☐ | 4☐ | 5☐ | 6☐ | 7☐ |
| 11.2. When I am accompanied at home | 1☐ | 2☐ | 3☐ | 4☐ | 5☐ | 6☐ | 7☐ |
| 11.3. When I am with friends | 1☐ | 2☐ | 3☐ | 4☐ | 5☐ | 6☐ | 7☐ |
| 11.4. In front of others | 1☐ | 2☐ | 3☐ | 4☐ | 5☐ | 6☐ | 7☐ |
| 11.5. In front of a recording | 1☐ | 2☐ | 3☐ | 4☐ | 5☐ | 6☐ | 7☐ |

## *ROLE OF MUSIC*

12. How important is music to you in your life?

*Not important* *Very important*

1☐ 2☐ 3☐ 4☐ 5☐ 6☐ 7☐

## *MUSIC-RELATED ACTIVITIES*

13. Do you do any musical activities (apart from music lessons at school)? For example: playing guitar, piano, musical theatre, playing in a music group...

YES ☐
NO ☐

If yes, what activity do you do?, and for how long have you been doing it?

a. ☐ Musical group __________years/months
b. ☐ Musical choir __________años/meses
c. ☐ Private lessons of any instrument or voice __________ years/months
d. ☐ Playing for fun by myself __________years/months
e. ☐ Playing for fun with friends __________years/months
f. ☐ Other: ________________________________________________________ years/months

- If the activity is not currently undertaken, has it been undertaken in the past?
